# Cloning and Expression Analysis of MEP Pathway Enzyme-encoding Genes in *Osmanthus fragrans*

**DOI:** 10.3390/genes7100078

**Published:** 2016-09-29

**Authors:** Chen Xu, Huogeng Li, Xiulian Yang, Chunsun Gu, Hongna Mu, Yuanzheng Yue, Lianggui Wang

**Affiliations:** 1 College of Landscape Architecture, Nanjing Forestry University, Nanjing 210037, China; xc127@foxmail.com (C.X.); yangxl339@sina.com (X.Y.); yuanzhengyue@163.com (Y.Y.); 2 Key Laboratory of Forest Genetics & Gene Engineering of the Ministry of Education, Nanjing Forestry University, Nanjing 210037, China; hgli@njfu.edu.cn; 3 Institute of Botany, Jiangsu Province and Chinese Academy of Sciences, Nanjing 210014, China; chunsungu@126.com; 4 College of Horticulture and Gardening, Yangtze University, Jingzhou 434025, China; hongnamu@163.com

**Keywords:** *Osmanthus fragrans*, MEP pathway, tissue-specific, flower development, diel oscillations

## Abstract

The 2-*C*-methyl-d-erythritol 4-phosphate (MEP) pathway is responsible for the biosynthesis of many crucial secondary metabolites, such as carotenoids, monoterpenes, plastoquinone, and tocopherols. In this study, we isolated and identified 10 MEP pathway genes in the important aromatic plant sweet osmanthus (*Osmanthus fragrans*). Multiple sequence alignments revealed that 10 MEP pathway genes shared high identities with other reported proteins. The genes showed distinctive expression profiles in various tissues, or at different flower stages and diel time points. The qRT-PCR results demonstrated that these genes were highly expressed in inflorescences, which suggested a tissue-specific transcript pattern. Our results also showed that *OfDXS1*, *OfDXS2*, and *OfHDR1* had a clear diurnal oscillation pattern. The isolation and expression analysis provides a strong foundation for further research on the MEP pathway involved in gene function and molecular evolution, and improves our understanding of the molecular mechanism underlying this pathway in plants.

## 1. Introduction

*Osmanthus fragrans*, also known as sweet osmanthus, sweet olive, and tea olive, is a traditional aromatic flowering tree that is native to China and has been cultivated for over 2500 years. It is considered to be one of top 10 Chinese traditional flowers and is also cultivated as an urban ornamental tree [1]. Owing to its pleasant aroma and evergreen properties, it is now widely distributed in Asian countries, such as China, Japan, Thailand, and India [2]. Today, 166 registered cultivars of *O*. *fragrans* have been classified into five groups based on morphological characteristics and growth habit. These are the Luteus group, the Albus group, the Aurantiacus group, the Asiaticus group, and the Colour group [3,4]. Generally, the cultivars in the Luteus group have golden-yellow flowers that only appear in the fall, whereas the Asiaticus group cultivars bloom all year round and have creamy-yellow flowers [5]. The fresh flowers are very abundant in aromatic compounds, including terpenoids, fatty acid derivatives, and phenylpropanoids/benzenoids [6,7]. Although the relative contents of the volatiles vary among different cultivars and developmental stages, the main aromatic components are the terpenoids, including monoterpenes ocimene and linalool [8,9,10]. Most of these substances are the primary components of perfumes and essential oils [11]. Because of the importance of these terpenoid compounds to the aesthetic value of *O. fragrans* plants, it has been of strong interest to understand their biosynthesis [12,13,14].

In plants, the biosynthesis of terpenoids is catalyzed by a family of enzymes collectively designated as terpene synthase (TPSs), which convert prenyl diphosphates to various subclasses of terpeneoids including monoterpenes [15]. Several *TPS* genes involved in the biosynthesis of volatile terpenoids from *O. fragrans* flowers have been isolated and characterized. The over-expressions of *OfTPS1*, *OfTPS2*, and *OfTPS3* in transgenic tobacco leaves results in the formation of the major monoterpenes, linalool and *trans*-β-ocimene [16]. In contrast to our knowledge about *TPS* genes, little is known about the biosynthesis of the substrates for TPSs, i.e., prenyl diphosphates. Generally, two biochemical pathways supply the prenyl diphosphates in plants: the mevalonate (MVA) pathway and the 2-*C*-methyl-d-erythritol 4-phosphate (MEP) pathway [17]. The MVA pathway functions in cytosol for the production of farnesyl diphosphate, which is the substrate for sesquiterpenes. In contrast, the MEP pathway is localized in plastids and produces geranyl diphosphate and geranylgeranyl diphosphate, which are substrates for monoterpenes and diterpenes, respectively [18]. Because the main terpenoids from *O. fragrans* flowers are monoterpenes, the MEP pathway is therefore of our interest for this study.

**Figure 1:**
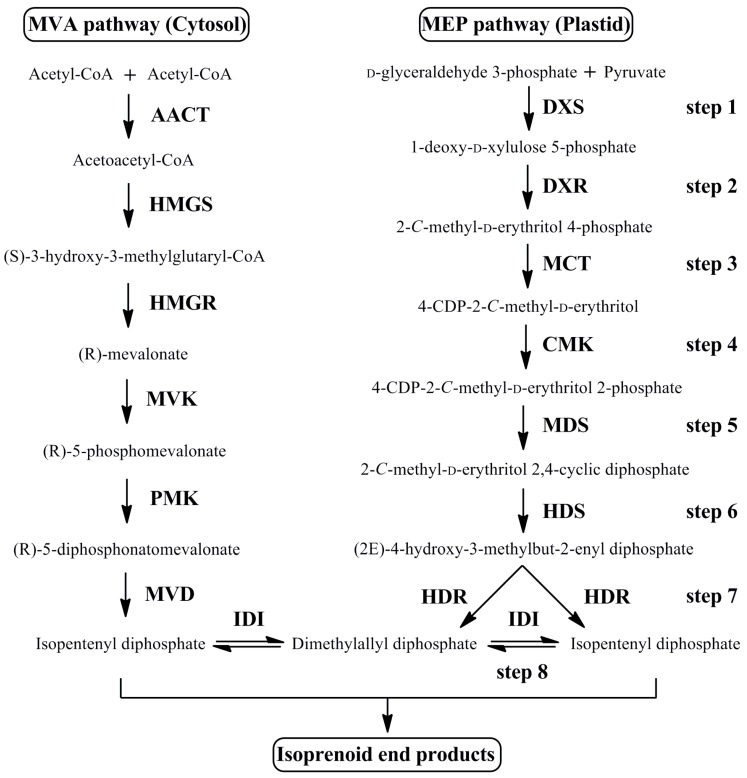
The steps of the MEP pathway leading to Isoprenoid biosynthetic. Enzymes of MEP pathway are as follows: step 1, DXS; step 2, DXR; step 3, MCT; step4, CMK; step 5, MDS; step 6, HDS; step 7, HDR; step 8, IDI.

The MEP pathway consists of eight enzymatic catalysis stages, and each step is schematically represented (Figure 1) [19,20]. This plastid-localized route begins with the production of 1-deoxy-d-xylulose 5-phosphate (DXP) by 1-deoxy-d-xylulose-5-phosphate synthase (DXS). The second step is catalyzed by 1-deoxy-d-xylulose-5-phosphate reductoisomerase (DXR), which transforms DXP to MEP [21]. Subsequently, MEP is converted to isopentenyl diphosphate (IPP) and dimethylallyl diphosphate (DMAPP) via 2-*C*-methyl-d-erythritol 4-phosphate cytidylyltransferase (MCT), 4-(cytidine 5′-diphospho)-2-*C*-methyl-d-erythritol kinase (CMK), 2-*C*-methyl-d-erythritol 2,4-cyclodiphosphate synthase (MDS), 4-hydroxy-3-methylbut-2-enyl diphosphate synthase (HDS), and 4-hydroxy-3-methylbut-2-enyl diphosphate reductase (HDR) [22]. The transformation between IPP and DMAPP proceeds through isopentenyl-diphosphate isomerase (IDI), which is a reversible reaction with crosstalk [23]. Apart from HDS and HDR, other crystal structures of enzymes in MEP route have been successfully represented [24].

The MEP pathway was originally detected in bacteria. However, further evidence has shown that it is widely found in phototrophic eukaryotes [25]. Various homologous genes have been isolated and cloned independently from many plant species, such as *Arabidopsis* (*Arabidopsis thaliana*) [26,27], periwinkle (*Catharanthus roseus*) [28,29], peppermint (*Mentha piperita*) [30,31], and tomato (*Lycopersicon esculentum*) [32,33]. Most enzymes in the MEP pathway are encoded by single genes in plants, including *Arabidopsis*, poplar (*Populus trichocarpa*), and rice (*Oryza sativa*) [24]. However, both DXS and HDR are reported to be encoded by a small gene family. For instance, there are three *DXS* genes encoding functional enzymes in maize (*Zea mays*), and two different genes encoding HDR have been identified in loblolly pine (*Pinus taeda*) [34,35]. Furthermore, previous studies have suggested that DXS and DXR have rate-limiting roles when controlling the metabolic flux through the MEP pathway [36]. Recently, the genetic transformation of *Artemisia annua* enhanced the biosynthesis of artemisinin by overexpressing *DXR* gene [37]. The metabolic engineering of plants is an effective way of improving desired characteristics, such as scent and color, which means research on MEP pathway enzyme-encoding genes is urgently needed because of their potential medical and industrial values [38].

In one of our recent studies, we analyzed the transcriptomes of *O*. *fragrans* by using Illumina technology. Many putative genes involved in floral scent biosynthesis were identified, including those of the MEP pathway [39]. The first objective of the present study is to isolate the full-length genes of the MEP pathway from *O. fragrans* and to compare them to the corresponding genes from other plant species. The second objective is to determine the expression patterns of the MEP pathway genes in order to understand their contribution to the biosynthesis of monoterpenes that are the major floral scent components of *O*. *fragrans*.

## 2. Materials and Methods

### 2.1. Plant Materials

**Figure 2:**
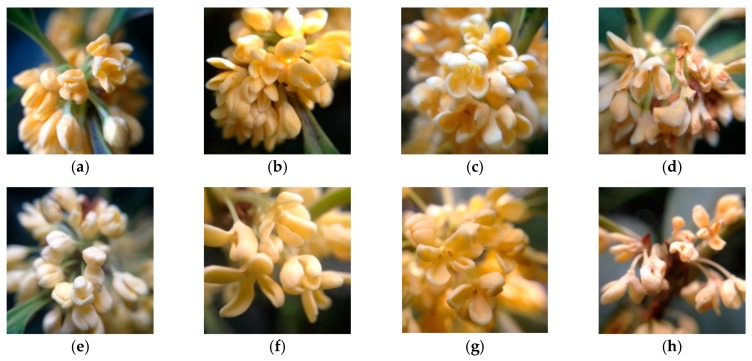
Flowering stages of “Boye Jingui” in (**a**) bud-eye stage (S1); (**b**) primary blooming stage (S2); (**c**) full blooming stage (S3); (**d**) flower fading stage (S4). Flowering stages of “Rixiang Gui” in (**e**) bud-eye stage (S1); (**f**) primary blooming stage (S2); (**g**) full blooming stage (S3); (**h**) flower fading stage (S4).

Two cultivars of *O*. *fragrans* “Boye Jingui” and *O*. *fragrans* “Rixiang Gui” were grown in the campus of Nanjing Forestry University in Jiangsu, China. Florets of cymose inflorescences (FCI) with the same anthesis were harvested at bud-eye stage (S1), primary blooming stage (S2), full blooming stage (S3), and flower fading stage (S4) in September 2014 (Figure 2). For tissue-specific gene expression studies, roots, stems and leaves, as well as florets of cymose inflorescences at full blooming stage were collected in September 2014. Materials used for diel analysis were collected every two hours (from 2:00 a.m. to 24:00 p.m.) at full blooming stage (S3) on 11 October 2015. All these samples were immediately frozen in liquid nitrogen and stored at −80 °C for further use.

### 2.2. Total RNA Extraction and Gene Cloning

The total RNA was obtained from the florets of cymose inflorescences of *O*. *fragrans* using RNAprep pure Kit (Tiangen Biotech, Beijing, China). The obtained RNA ratio of A260/280 was quantified by NanoDrop 2000 Spectrophotometer (Thermo Scientific, Waltham, MA, USA). The RNA integrity was evaluated by 1.5% agarose gel electrophoresis. Then the first strand cDNA reaction with 1 μg total RNA was performed using RevertAid First Strand cDNA Synthesis Kit (Thermo Scientific). According to the MEP pathway unigene sequences from the transcriptomic data of *O. fragrans*, the specific primers were designed to clone *OfDXS1*, *OfDXS2*, *OfDXR*, *OfMCT*, *OfCMK*, *OfMDS*, *OfHDS*, *OfHDR1*, *OfHDR2*, and *OfIDI* (Table S1). By using LA Taq (Takara Biotechnology, Dalian, China), purified DNA fragments of polymerase chain reaction (PCR) were ligated into pEASY^®^-T1 cloning vector (Transgen Biotech, Beijing, China) and transformed into *E*. *coli* chemically competent cells. Positive recombinant clones were identified and sequenced using the universal M13 primers.

### 2.3. Cloning of Full Length Genes by RACE

Rapid amplification of cDNA ends (RACE) was used to obtain the 3′ ends and 5′ ends of target genes according to the manufacture’s procedure (Takara Biotechnology). The specific primers for 3′ RACE and 5′ RACE were designed using Oligo 6.0 software based on the obtained partial sequences. The primer sequences and PCR conditions were listed (Table S2). By sequential nested PCR, these unknown regions were amplified and sequenced. Then full-length genes were assembled together by the Lasergene 7.0 software (Dnastar, Madison, WI, USA). The coding regions were confirmed by PCR detection from start codon to stop codon.

### 2.4. Gene Expression Analysis

Following the MIQE guidelines, the primers of target genes for quantitative real-time PCR (qRT-PCR) were selected using primer premier 5.0 software (Premier biosoft, Palo Alto, CA, USA) (Table S3), and the absence of hairpin structure and primer dimer were predicted by Oligo 6.0 software (Molecular biology insights, Colorado Springs, CO, USA) [40]. Total RNA preparation was performed as described previously according to the manufacturer's instructions. Then first strand cDNA was synthesized from 1 μg total RNA and diluted five-fold for gene expression experiment. The qRT-PCR experiment was carried out by using an ABI StepOnePlus Systems (Applied Biosystems, Carlsbad, CA, USA) and SYBR Premix Ex Taq (Takara Biotechnology). The PCR conditions were as follows: 95 °C for 30 s, followed by 40 cycles of 95 °C for 5 s, and 58 °C for 30 s. The qRT-PCR for each sample was repeated three times. Previous validated genes *OfRAN*, *OfRPB2*, and *OfACT* were used as internal normalizations for different organs, different flowering stages and diel variations, respectively [12]. Each primer pair was validated the specificity by melt curve analysis, and the gene expression levels were calculated by the 2^−ΔΔCT^ method. The qRT-PCR results were analyzed by using ABI StepOne software (Applied Biosystems).

## 3. Results

### 3.1. Cloning and Sequence Analysis of MEP Pathway Genes

To clone *OfDXS1*, *OfDXS2*, *OfDXR*, *OfMCT*, *OfCMK*, *OfMDS*, *OfHDS*, *OfHDR1*, *OfHDR2*, and *OfIDI*, degenerate primers were designed to obtain 2232 bp, 1085 bp, 1453 bp, 596 bp, 1306 bp, 628 bp, 2366 bp, 359 bp, 604 bp, and 669 bp sized amplicons respectively. Based on the partial gene sequences, the 3′ region and 5′ region were amplified and sequenced by RACE. The size of full-length cDNA, open reading frame (ORF), amino acids, molecular weight, and isoelectric point (pI) were listed (Table 1). We submitted the 10 sweet osmanthus full-length cDNAs of MEP genes to NCBI GenBank with the accession number KX400841–KX400850.

The amino acid sequences of 10 MEP genes were aligned with other plants to reveal identities and conserved domains in the NCBI database. Multiple alignments showed that the identities ranged from 73% to 92%, in which *OfDXS1*, *OfDXR*, *OfHDS*, and *OfIDI* shared the higher identities between 88% and 92%, *OfMCT*, *OfCMK*, and *OfMDS* shared the lower identities between 73% and 82%, and *OfDXS2* had the intermediate identity between 85% and 87%. Both *OfDXS1* and *OfDXS2* contained three conserved domains, a thiamine diphosphate-dependent domain at the N-terminus, a pyrimidine binding domain at the medial position, and a transketolase domain at the C-terminus. *OfDXR* also contained three reductoisomerase domains, which was located at the position 80–208 aa, 222–305 aa, and 337–459 aa. *OfMCT* had an IspD domain at the C-terminus, which is also known as ygbP domain. *OfCMK* contained two GHMP kinase domains at the position 176–234 aa and 287–360 aa. *OfMDS* showed a trimer YgbB domain at the position 76–230 aa. Conserved domain of GcpE was found in *OfHDS* at the position 89–731 aa. In *OfHDR1* and *OfHDR2*, LytB domain was shown at the C-terminus. *OfIDI* showed a NUDIX hydrolase domain at the position 53–205 aa. The amino acid sequences among MEP proteins were composed of multiple conserved residues, which were crucial to form distinct dimensional structures and specific biological functions (Table S4).

### 3.2. Expression Analysis of MEP Genes in Different Organs

**Figure 3:**
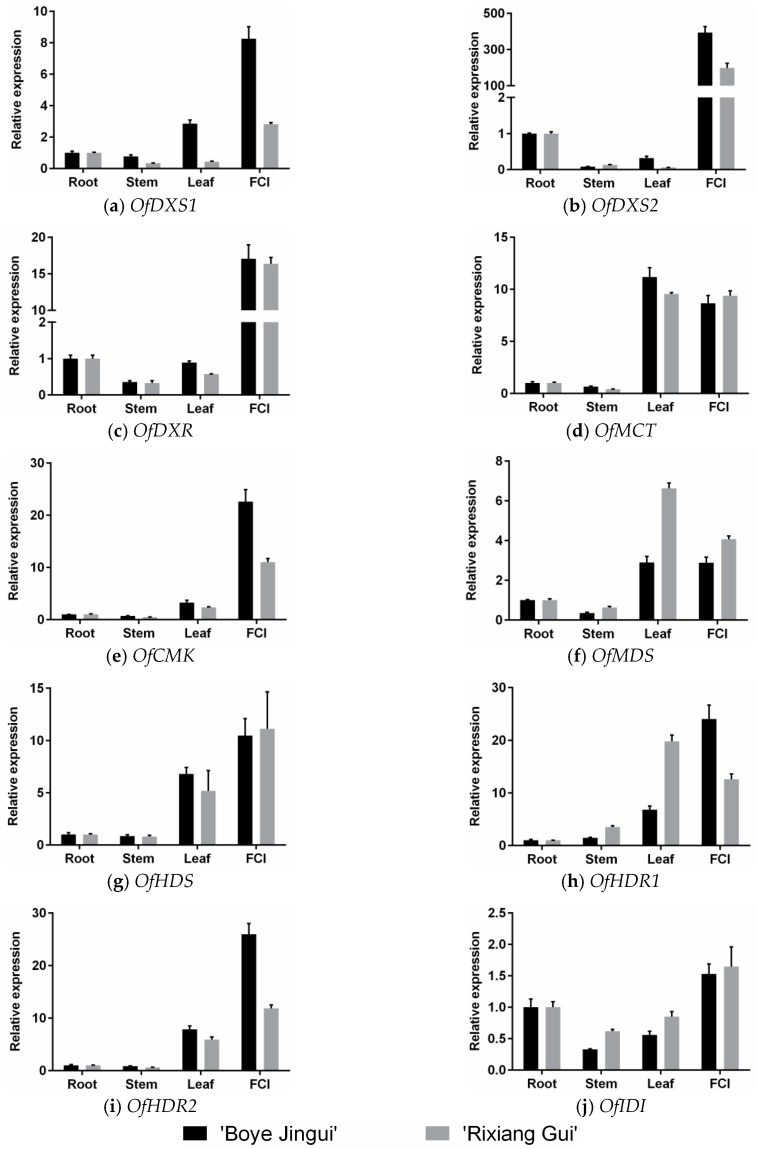
Expression patterns of MEP pathway genes in four different organs of *Osmanthus fragrans*. FCI: florets of cymose inflorescences at full blooming stage. Data were presented as means with error bars indicating standard deviation.

To investigate the tissue-specific expressions of the MEP genes, the transcript levels of two *O*. *fragrans* cultivars, “Boye” and “Rixiang”, were detected in different organs including roots, stems, leaves, and inflorescences by using qRT-PCR. The transcript levels of *OfDXS1*, *OfDXS2*, *OfDXR*, *OfMCT*, *OfCMK*, *OfMDS*, *OfHDS*, *OfHDR1*, *OfHDR2*, and *OfIDI* were measured (Figure 3).

The tissue-specific results suggested that *OfDXS1*, *OfDXS2*, *OfDXR*, *OfCMK*, and *OfHDR2* expressions were significantly abundant in the inflorescences, compared with other organs. In cultivar “Boye”, the *OfDXS1* and *OfCMK* transcript levels in the inflorescences were almost 8-fold and 22-fold higher than that in the roots respectively, while in cultivar “Rixiang” they were only 2.8-fold and 11-fold, respectively. In both cultivar “Boye” and “Rixiang”, *OfDXR* showed over 15-fold transcript levels in the inflorescences than in the roots. As for *OfDXS2*, the inflorescences were found to contain the highest transcript level. In cultivar “Boye”, the *OfDXS2* transcript level in the inflorescences was virtually 427-fold higher than that in the roots, whereas in cultivar “Rixiang” it was merely 224-fold. For cultivar “Boye” and “Rixiang”, *OfMCT*, *OfMDS*, *OfHDS*, and *OfHDR1* showed higher transcript levels in the leaves and the inflorescences than in the roots and the stems. In addition, the *OfMCT* transcript levels were paralleled in the leaves and the inflorescences. In cultivar “Boye”, *OfHDS* and *OfHDR1* showed higher transcript levels in the inflorescences than in the rest of the organs. Whereas in cultivar “Rixiang”, *OfMDS* and *OfHDR1* showed higher transcript levels in the leaves. Furthermore, *OfIDI* showed slightly different transcript profiles among the four organs, which were consistent in the two cultivars. The *OfIDI* transcript level in the petals was 1.5-fold higher than that in the roots, and almost 3-fold higher than that in the stems or the leaves.

### 3.3. Expression Analysis of MEP Genes Over Flower Development

**Figure 4:**
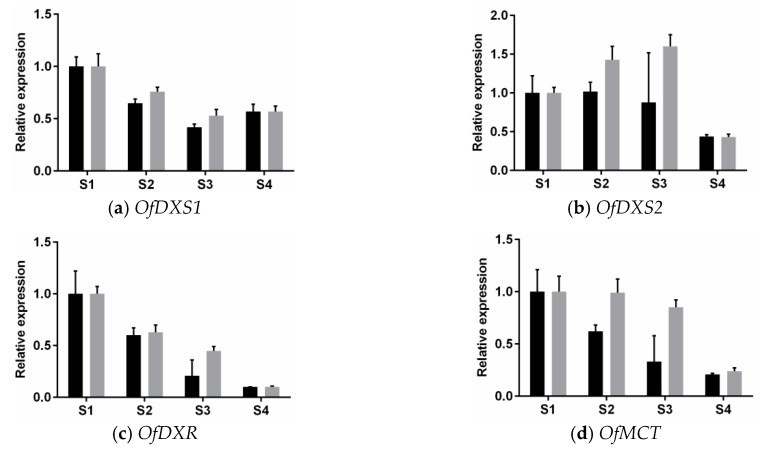

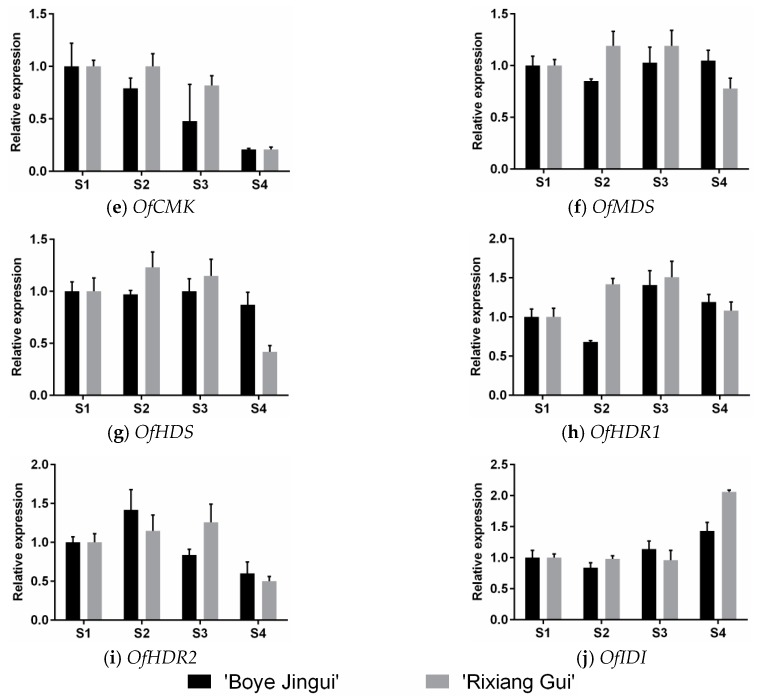
Expression patterns of MEP pathway genes at four different flowering stages of *Osmanthus fragrans*. These cDNA templates were isolated from bud-eye stage (S1), primary blooming stage (S2), full blooming stage (S3), flower fading stage (S4). Data were presented as means with error bars indicating standard deviation.

To determine the expression patterns during flower development, qRT-PCR were conducted to detect the transcript levels of MEP genes at four flowering stages, including bud-eye stage (S1), primary blooming stage (S2), full blooming stage (S3), and flower fading stage (S4) (Figure 4).

The experimental results showed the MEP genes were all detected at four flowering stages, however their transcript patterns varied from each other. For *OfDXS1*, the transcripts in “Boye” and “Rixiang” both displayed downregulated trends coincidently during the first three stages. The *OfDXS2* transcript levels showed entirely opposite trends in the two cultivars: in “Boye” high transcript level was maintained in the first three stages, whereas in “Rixiang”, it first increased steadily from S1 to S3 stage, and then declined sharply at S4 stage. The *OfDXR* transcript levels declined constantly in the two cultivars from S1 to S4 stage. For *OfMCT* and *OfCMK*, their transcript levels in “Boye” showed a regularly downregulated trend at the four stages. However, their transcripts in “Rixiang” remained at a high level from S1 to S3 stage, and then decreased considerably from S3 to S4 stage. For *OfMDS* and *OfHDS*, their transcript levels in “Boye” did not show a significant change at the four stages. However, their transcript levels in “Rixiang” showed a slight rise from S1 to S2 stage, and then declined from the S3 to S4 stage. For *OfHDR1* and *OfHDR2*, their transcript levels in “Rixiang” showed a similar profile with that of *OfMDS* and *OfHDS*, while their transcript levels in “Boye” showed a reverse trend from S1 to S2 stage. For *OfIDI*, the transcripts in “Boye” and “Rixiang” maintained almost identical levels during the first three stages, but ascended to 1.25-fold and 2.15-fold from S3 to S4 stage, respectively.

### 3.4. Expression analysis of MEP Genes during Diel Oscillations

**Figure 5:**
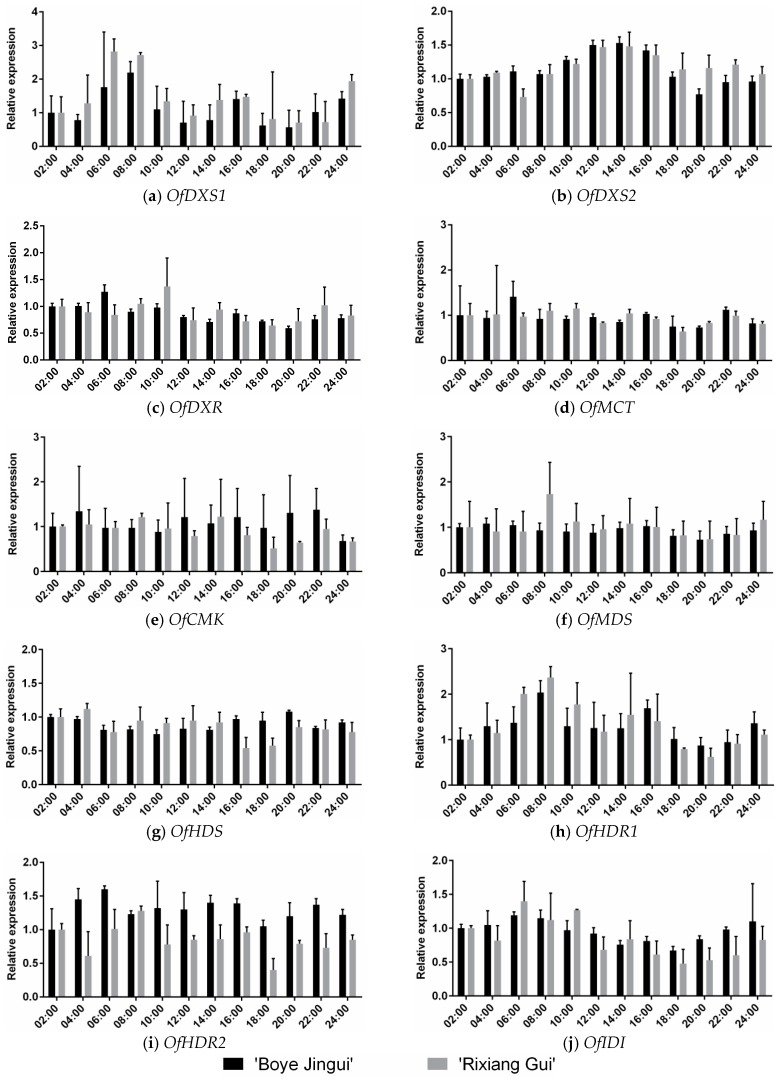
Expression patterns of MEP pathway genes during diel oscillations of *Osmanthus fragrans*. These cDNA templates were isolated from 02:00 a.m. to 24:00 p.m. with two-hour intervals in full blooming stage. Data were presented as means with error bars indicating standard deviation.

To further study the expression patterns during diel oscillations from sweet osmanthus flowers over time, we chose 12 sampling time points with two-hour intervals in full blooming stage for daily analysis. Using qRT-PCR, the transcript levels of the 10 MEP genes were detected in the two cultivars (Figure 5).

Each of the MEP genes showed a particular oscillating pattern during the daytime and night cycles. In cultivar “Boye” and “Rixiang”, the *OfDXS1* transcript levels both exhibited a clear peak in the morning. The *OfDXS2* transcript levels showed a typical diurnal oscillation both in cultivars “Boye” and “Rixiang”, which escalated in the morning, reached the peak at midday, then de-escalated in the afternoon. For *OfDXR*, the transcript level in “Boye” showed a slight peak at 06:00 h, whereas peak transcript of “Rixiang” appeared to a later time point (10:00 h). The *OfMCT* transcript in “Boye” achieved the highest level at 06:00 h, while in “Rixiang” slight oscillations occurred during the whole day. Compared with other time points, the *OfCMK* transcript in “Boye” maintained higher level from 12:00 to 22:00 h. Yet in “Rixiang”, the *OfCMK* transcript level decreased after 14:00 h. For *OfMDS*, the transcript level in “Boye” oscillated steadily during the whole day, whereas the transcript level in “Rixiang” revealed a significant peak in the morning with 1.73-fold higher than the corresponding predawn level. In “Boye”, the *OfHDS* transcript peaked to 1.5-fold higher levels at 04:00, 14:00, and 20:00 h, while significant oscillation was undetected in “Rixiang”. For *OfHDR1*, the transcript levels in “Boye” and “Rixiang” firstly crested at 08:00 h, thereafter reaching another peak at 14:00 and 16:00 h, respectively. While the *OfHDR2* transcript levels showed a slight peak between 06:00 and 08:00 h in the two cultivars. As for *OfIDI*, the transcript levels showed a gradual decline in the daytime.

## 4. Discussion

The MEP pathway genes have been isolated and identified in a number of plant species, including *Arabidopsis* [26,27], peppermint [30,31], tomato [32,33], and rice [24]. However, this pathway has not yet been studied in sweet osmanthus. In this study, gene cloning allows the analysis of the MEP pathway genes sequences in sweet osmanthus and the results will facilitate further research on gene function and molecular evolution.

### 4.1. The MEP Pathway Genes of O. fragrans Are Highly Related to Those from Other Plants

The MEP pathway contains eight enzymatic steps and previous research has shown that terpenoids biosynthesis is regulated by a series of structural and functional genes. [24]. DXS, the first committed enzyme in the MEP pathway, influencing the accumulation of downstream isoprenoids, is encoded by a small multigene family in higher plants. In this study, we successfully isolated two *OfDXS* genes from sweet osmanthus. In the second enzymatic step of the MEP pathway, DXR also has a rate-limiting effect on the accumulation of MEP-derived isoprenoids [41,42]. Furthermore, the biosynthesis of MEP limits the production of downstream isoprenoids in *Arabidopsis* [43]. In the third step of the MEP pathway, AtMCT contain a plastid targeting sequence in *Arabidopsis* [44]. The *CMK* genes, which contain putative ATP binding sites and plastid target sequences, were also cloned from tomato and peppermint [45]. The GbMDS from ginkgo biloba is well conserved in the protein family and highly similar (over 70% identity) to other plants [46]. Although less is known about *HDS* and *HDR* than other genes in the MEP pathway, it has been shown that HDR is encoded by muticopy genes in plants, and we obtained two *OfHDR* genes from sweet osmanthus [35]. The last step in the MEP pathway is an isomerization reaction, which is catalyzed by IDI, and is also a rate-limiting step during isoprenoid synthesis [47].

In this study, through the efforts of transcriptome mining and RACE, we successfully obtained 10 full-length MEP pathway cDNAs from sweet osmanthus. These included *OfDXS1*, *OfDXS2*, *OfDXR*, *OfMCT*, *OfCMK*, *OfMDS*, *OfHDS*, *OfHDR1*, *OfHDR2*, and *OfIDI*. Their deduced protein sequences were all highly similar to those of other plants. Interestingly, the sequence alignments of *OfDXS1*, *OfDXS2*, *OfDXR*, *OfMCT*, *OfCMK*, *OfHDS*, *OfHDR1*, and *OfHDR2* showed 92%, 87%, 91%, 80%, 82%, 92%, 86%, and 88% identity with reported corresponding proteins from *Sesamum indicum* respectively, which suggested that there was an evolutionary conserved relationship between sweet osmanthus and *S*. *indicum*. However, OfMDS shared a high similarity with *Salvia miltiorrhiza*. By bioinformatics analysis, we found that the MEP genes were conserved over their protein sequences, but their detailed evolutionary relationships need further investigation.

### 4.2. Expression Patterns of the MEP Pathway Genes Suggest that Enhanced Biosynthesis of Substrate Contributes to the Production of Monoterpenes in O. fragrans Flowers

The biosynthesis of terpenoids can be regulated at, at least, two levels: the level of terpene synthases and the level of the substrates of TPSs. While a previous study has shown the importance of the regulation of *TPS* genes [16], the present study indicates the importance of the regulation of the substrate biosynthesis.

The transcript results showed that the MEP genes were all highly expressed in the inflorescences compared with other organs. This suggested that there was a tissue-specific expression profile among these genes, which led to the biosynthesis of specific downstream isoprenoid-derived products [24]. Similarly, it has been reported that terpene synthase (*TPS*) genes involved in volatile terpenoid synthesis have been cloned and shown to be flower-specific in *Clarkia breweri* [48], *Antirrhinum majus* [49], and *A*. *thaliana* [50]. *OfDXS2*, which is involved in the first step of the MEP pathway, showed a clear flower-specific transcript profile and its transcript level was several hundred-fold higher than in the roots. It has been suggested that the floral and vegetative tissues are the main scent sources in many plants [51]. Therefore, the enormous expression might lead to the specific accumulation of monoterpenes and sesquiterpenes in flowers [52].

Previous studies have shown that the pigment and essential oil compositions vary in *O*. *fragrans* floral developmental process [9,53]. Furthermore, previous research has suggested that the aromatic compounds and relative contents differ among *O*. *fragrans* cultivar groups, including cultivars “Boye” and “Rixiang” [54]. In this study, the expression profiles of these MEP pathway genes were investigated at different developmental stages by qRT-PCR. Notably, *OfDXS1* and *OfDXS2* remained continuous high expression during the anthesis, as well as *OfMDS*, *OfHDS*, and *OfHDR1*. These results were consistent with the expression level of *OfDXS* in “Yanhong Gui”, where there was a substantial accumulation of α- and β-carotene [1]. Moreover, carotenoids are considered to be the crucial substrates for the OfCCD1 enzyme, which produces α- and β-ionone aroma compounds in flowers of *O*. *fragrans* [8]. In contrast, the *OfDXR* expression level was down-regulated dramatically from S1 to S4 stage. Interestingly, the *OfMCT* and *OfCMK* expression patterns in “Boye” and “Rixiang” were not the same. These variations in expression might be caused by cultivar differences.

### 4.3. Expression Patterns of MEP Pathway Genes during Diel Oscillations

It has been observed that the MEP pathway genes fluctuated rhythmically over a daily light/dark cycle [55]. In *A*. *thaliana*, the expression of MEP pathway genes was reported to be controlled by light [56]. However, in snapdragon flowers, MEP gene expressions follows a diurnal rhythm, which is regulated by an endogenous circadian clock [57]. Recent study has shown that in many plants, scent emission can be regulated either by circadian clock or by light, mostly by the gene expression levels [58].

Previous research has suggested that the flower volatile emissions of sweet osmanthus follow a diurnal pattern, and sweet osmanthus flowers release the highest total amount of volatiles at 10:00 h in the daytime [2]. In this study, we monitored the transcript levels of the MEP pathway genes every 2 h for 24 h at the *O*. *fragrans* full blooming stage. The *OfDXS2* expression level, not *OfDXR*, showed a diurnal oscillation profile that increased under light conditions with the highest accumulation occurring between 12:00 and 14:00 h. Similarly, the transcript levels of *OfDXS1* and *OfHDR1* showed considerable intraday variations with a clear peak in the morning. Apart from *OfDXS1*, *OfDXS2*, and *OfHDR1*, the rest of the MEP pathway genes lacked obvious diurnal oscillation patterns. Although previous mathematical modelling data suggested that flux through the MEP pathway is due to the photosynthesis-dependent supply of metabolic substrates, additional experimental work is needed to clarify the contribution of enzymatic substrate biosynthesis to diurnal patterns of volatile emission [59].

## 5. Conclusions

In this study, the genes in the MEP pathway in *O. fragrans* were cloned, compared with those of other plants, and analyzed for their expression patterns. The sequence alignment analysis revealed that the MEP pathway genes of *O. fragrans* had high sequence identities with other reported proteins, which suggests that an evolutionarily conserved relationship exists. The qRT-PCR results showed that many MEP pathway genes had a higher-level of expression in the inflorescences, supporting that the enhanced production of the prenyl diphosphates—the substrates of terpene synthases—contributes to the biosynthesis of monoterperne floral volatiles. In addition, the expressions of several genes, such as *OfDXS1*, *OfDXS2*, and *OfHDR1*, exhibited a diurnal oscillation pattern. Our results lay an important foundation for future research on functional and molecular evolutionary analysis of the terpene pathway genes involved in the production of terpene floral volatiles in *O. fragrans*.

## Figures and Tables

**Table 1:** Sequence characteristics of 10 MEP pathway genes in *Osmanthus fragrans*.

Gene	Accession No.	Full Length (bp)	ORF (bp)	Amino Acids (aa)	Molecular Weight (kDa)	PI
*OfDXS1*	KX400841	2645	2172	723	78.1	6.91
*OfDXS2*	KX400842	2533	2148	715	76.9	6.91
*OfDXR*	KX400843	1687	1425	474	51.3	6.04
*OfMCT*	KX400844	1155	939	312	34.4	7.67
*OfCMK*	KX400845	1543	1206	401	44.4	5.75
*OfMDS*	KX400846	991	702	233	25.1	8.64
*OfHDS*	KX400847	2551	2229	742	82.5	5.78
*OfHDR1*	KX400848	1642	1386	461	52.0	5.51
*OfHDR2*	KX400849	1593	1380	459	51.8	5.73
*OfIDI*	KX400850	1130	708	235	26.9	5.14

## Supplementary Materials

The following are available online at www.mdpi.com/2073-4425/7/10/78. Table S1: Primers used for partial cDNA cloning; Table S2: Primers used for RACE reaction; Table S3: Primers used for qRT-PCR; Table S4: Conserved domain analysis for MEP genes.

## Abbreviations

The following abbreviations are used in this manuscript:

DXS: 1-deoxy-d-xylulose-5-phosphate synthase
DXR: 1-deoxy-d-xylulose-5-phosphate reductoisomerase
MCT: 2-*C*-methyl-d-erythritol 4-phosphate cytidylyltransferase
CMK: 4-(cytidine 5′-diphospho)-2-*C*-methyl-d-erythritol kinase
MDS: 2-*C*-methyl-d-erythritol 2,4-cyclodiphosphate synthase
HDS: 4-hydroxy-3-methylbut-2-enyl diphosphate synthase
HDR: 4-hydroxy-3-methylbut-2-enyl diphosphate reductase
IDI: isopentenyl-diphosphate isomerase

## References

[1] Zhang C., Wang Y., Fu J., Bao Z., Zhao H. (2016). Transcriptomic analysis and carotenogenic gene expression related to petal coloration in *Osmanthus fragrans* ‘Yanhong Gui’. Trees.

[2] Baldermann S., Kato M., Kurosawa M., Kurobayashi Y., Fujita A., Fleischmann P., Watanabe N. (2010). Functional characterization of a carotenoid cleavage dioxygenase 1 and its relation to the carotenoid accumulation and volatile emission during the floral development of *Osmanthus fragrans* Lour.. J. Exp. Bot..

[3] Xiang Q.B., Liu Y.L. (2008). Classification system of sweet osmanthus cultivars. An Illustrated Monograph of the Sweet Osmanthus Cultivars in China.

[4] Yuan W.J., Li Y., Ma Y.F., Han Y.J., Shang F.D. (2015). Isolation and characterization of microsatellite markers for *Osmanthus fragrans* (Oleaceae) using 454 sequencing technology. Genet. Mol. Res..

[5] Han Y.J., Chen W.C., Yang F.B., Wang X.H., Dong M.F., Zhou P., Shang F.D. (2015). cDNA-AFLP analysis on 2 *Osmanthus fragrans* cultivars with different flower color and molecular characteristics of *OfMYB1* gene. Trees.

[6] Deng C.H., Song G.X., Hu Y.M. (2004). Application of HS-SPME and GC-MS to characterization of volatile compounds emitted from osmanthus flowers. Ann. Chim..

[7] Cai X., Mai R.Z., Zou J.J., Zhang H.Y., Zeng X.L., Zheng R.R., Wang C.Y. (2014). Analysis of aroma-active compounds in three sweet osmanthus (*Osmanthus fragrans*) cultivars by GC-olfactometry and GC-MS. J. Zhejiang Univ. B.

[8] Baldermann S., Kato M., Fleischmann P., Watanabe N. (2012). Biosynthesis of α- and β-ionone, prominent scent compounds, in flowers of *osmanthus fragrans*. Acta Biochim. Pol..

[9] Wang L.M., Li M.T., Jin W.W., Li S., Zhang S.Q., Yu L.J. (2009). Variations in the components of *Osmanthus fragrans* Lour. essential oil at different stages of flowering. Food Chem..

[10] Xin H.P., Wu B.H., Zhang H.H., Wang C.Y., Li J.T., Yang B., Li S.H. (2013). Characterization of volatile compounds in flowers from four groups of sweet osmanthus (*Osmanthus fragrans*) cultivars. Can. J. Plant Sci..

[11] Lei G.M., Mao P.Z., He M.Q., Wang L.H., Liu X.S., Zhang A.Y. (2016). Water-soluble essential oil components of fresh flowers of *Osmanthus fragrans* Lour.. J. Essent. Oil Res..

[12] Zhang C., Fu J., Wang Y., Bao Z., Zhao H. (2015). Identification of suitable reference genes for gene expression normalization in the quantitative real-time PCR analysis of sweet osmanthus (*Osmanthus fragrans* Lour.). PLoS ONE.

[13] Moronkola D.O., Aiyelaagbe O.O., Ekundayo O. (2005). Syntheses of eight fragrant terpenoids [ionone derivatives] via the aldol condensation of citral and eight ketones. J. Essent. Oil Bear. Plants.

[14] Muhlemann J.K., Klempien A., Dudareva N. (2014). Floral volatiles: From biosynthesis to function. Plant Cell Environ..

[15] Tholl D. (2006). Terpene synthases and the regulation, diversity and biological roles of terpene metabolism. Curr. Opin. Plant Biol..

[16] Zeng X.L., Liu C., Zheng R.R., Cai X., Luo J., Zou J.J., Wang C.Y. (2016). Emission and accumulation of monoterpene and the key terpene synthase (TPS) associated with monoterpene biosynthesis in *Osmanthus fragrans* Lour.. Front. Plant Sci..

[17] Lichtenthaler H.K. (1999). The 1-deoxy-d-xylulose-5-phosphate pathway of isoprenoid biosynthesis in plants. Annu. Rev. Plant Physiol. Plant Mol. Biol..

[18] Gutensohn M., Orlova I., Nguyen T.T.H., Davidovich-Rikanati R., Ferruzzi M.G., Sitrit Y., Lewinsohn E., Pichersky E., Dudareva N. (2013). Cytosolic monoterpene biosynthesis is supported by plastid-generated geranyl diphosphate substrate in transgenic tomato fruits. Plant J..

[19] Singh H., Gahlan P., Kumar S. (2013). Cloning and expression analysis of 10 genes associated with picrosides biosynthesis in *Picrorhiza kurrooa*. Gene.

[20] Pulido P., Perello C., Rodríguez-Concepción M. (2012). New insights into plant isoprenoid metabolism. Mol. Plant.

[21] Lichtenthaler H. (2000). Non-mevalonate isoprenoid biosynthesis: Enzymes, genes and inhibitors. Biochem. Soc. Trans..

[22] Rohdich F., Kis K., Bacher A., Eisenreich W. (2001). The non-mevalonate pathway of isoprenoids: Genes, enzymes and intermediates. Curr. Opin. Chem. Biol..

[23] Hunter W.N. (2007). The non-mevalonate pathway of isoprenoid precursor biosynthesis. J. Biol. Chem..

[24] Cordoba E., Salmi M., León P. (2009). Unravelling the regulatory mechanisms that modulate the MEP pathway in higher plants. J. Exp. Bot..

[25] Rohmer M. (1999). The discovery of a mevalonate-independent pathway for isoprenoid biosynthesis in bacteria, algae and higher plants. Nat. Prod. Rep..

[26] Estévez J.M., Cantero A., Romero C., Kawaide H., Jiménez L.F., Kuzuyama T., Seto H., Kamiya Y., León P. (2000). Analysis of the expression of *CLA1*, a gene that encodes the 1-deoxyxylulose 5-phosphate synthase of the 2-*C*-methyl-d-erythritol-4-phosphate pathway in *Arabidopsis*. Plant Physiol..

[27] Carretero-paulet L., Ahumada I., Cunillera N., Rodríguez-Concepción M., Ferrer A., Boronat A., Campos N. (2002). Expression and molecular analysis of the Arabidopsis *DXR* Gene encoding 1-deoxy-d-xylulose 5-phosphate reductoisomerase, the first committed enzyme of the 2-*C*-methyl-d-erythritol 4-phosphate pathway. Plant Physiol..

[28] Chahed K., Oudin A., Guivarc H.N., Hamdi S., Chénieux J.C., Rideau M., Clastre M. (2000). 1-deoxy-d-xylulose 5-phosphate synthase from periwinkle: cDNA identification and induced gene expression in terpenoid indole alkaloid-producing cells. Plant Physiol. Biochem..

[29] Veau B., Courtois M., Oudin A., Chénieux J.C., Rideau M., Clastre M. (2000). Cloning and expression of cDNAs encoding two enzymes of the MEP pathway in *Catharanthus roseus*. Biochim. Biophys. Acta.

[30] Lange B.M., Wildung M.R., McCaskill D., Croteau R. (1998). A family of transketolases that directs isoprenoid biosynthesis via a mevalonate-independent pathway. Proc. Natl. Acad. Sci. USA.

[31] Lange B.M., Croteau R. (1999). Isoprenoid biosynthesis via a mevalonate-independent pathway in plants: Cloning and heterologous expression of 1-deoxy-d-xylulose-5-phosphate reductoisomerase from peppermint. Arch. Biochem. Biophys..

[32] Lois L.M., Rodríguez-Concepción M., Gallego F., Campos N., Boronat A. (2000). Carotenoid biosynthesis during tomato fruit development: Regulatory role of 1-deoxy-d-xylulose 5-phosphate synthase. Plant J..

[33] Rohdich F., Wungsintaweekul J., Lüttgen H., Fischer M., Eisenreich W., Schuhr C.A., Fellermeier M., Schramek N., Zenk M.H., Bacher A. (2000). Biosynthesis of terpenoids: 4-diphosphocytidyl-2-*C*- methyl-d-erythritol kinase from tomato. Proc. Natl. Acad. Sci. USA.

[34] Cordoba E., Porta H., Arroyo A., San Román C., Medina L., Rodríguez-Concepción M., León P. (2011). Functional characterization of the three genes encoding 1-deoxy-d-xylulose 5-phosphate synthase in maize. J. Exp. Bot..

[35] Kim S.M., Kuzuyama T., Kobayashi A., Sando T., Chang Y.J., Kim S.U. (2008). 1-hydroxy-2-methyl-2-(E)-butenyl 4-diphosphate reductase (IDS) is encoded by multicopy genes in gymnosperms *Ginkgo biloba* and *Pinus taeda*. Planta.

[36] Tong Y.R., Su P., Zhao Y.J., Zhang M., Wang X.J., Liu Y.J., Zhang X.N., Gao W., Huang L.Q. (2015). Molecular cloning and characterization of *DXS* and *DXR* genes in the terpenoid biosynthetic pathway of *Tripterygium wilfordii*. Int. J. Mol. Sci..

[37] Xiang L., Zeng L., Yuan Y., Chen M., Wang F., Liu X., Zeng L., Lan X., Liao Z. (2012). Enhancement of artemisinin biosynthesis by overexpressing *dxr*, *cyp71av1* and *cpr* in the plants of *Artemisia annua* L.. Plant Omics.

[38] Roberts S.C. (2007). Production and engineering of terpenoids in plant cell culture. Nat. Chem. Biol..

[39] Mu H.N., Li H.G., Wang L.G., Yang X.L., Sun T.Z., Xu C. (2014). Transcriptome sequencing and analysis of sweet osmanthus (*Osmanthus fragrans* Lour.). Genes Genomics.

[40] Bustin S.A., Benes V., Garson J.A., Hellemans J., Huggett J., Kubista M., Mueller R., Nolan T., Pfaffl M.W., Shipley G.L. (2009). The MIQE guidelines: Minimum information for publication of quantitative real-time PCR experiments. Clin. Chem..

[41] Yan X.M., Zhang L., Wang J., Liao P., Zhang Y., Zhang R., Kai G.Y. (2009). Molecular characterization and expression of 1-deoxy-d-xylulose 5-phosphate reductoisomerase (*DXR*) gene from *Salvia miltiorrhiza*. Acta Physiol. Plant.

[42] Munos J.W., Pu X., Mansoorabadi S.O., Kim H.J., Liu H.W. (2009). A secondary kinetic isotope effect study of the 1-deoxy-d-xylulose-5-phosphate reductoisomerase-catalyzed reaction: Evidence for a retroaldol-aldol rearrangement. J. Am. Chem. Soc..

[43] Carretero-Paulet L., Cairó A., Botella-Pavía P., Besumbes O., Campos N., Boronat A., Rodríguez-Concepción M. (2006). Enhanced flux through the methylerythritol 4-phosphate pathway in *Arabidopsis* plants overexpressing deoxyxylulose 5-phosphate reductoisomerase. Plant Mol. Biol..

[44] Rohdich F., Wungsintaweekul J., Eisenreich W., Richter G., Schuhr C.A., Hecht S., Zenk M.H., Bacher A. (2000). Biosynthesis of terpenoids: 4-diphosphocytidyl-2*C*-methyl-d-erythritol synthase of *Arabidopsis thaliana*. Proc. Natl. Acad. Sci. USA.

[45] Dubey V.S., Bhalla R., Luthra R. (2003). An overview of the non-mevalonate pathway for terpenoid biosynthesis in plants. J. Biosci..

[46] Kim S.M., Kuzuyama T., Chang Y.J., Kim S.U. (2006). Cloning and characterization of 2-*C*-methyl-d-erythritol 2,4-cyclodiphosphate synthase (*MECS*) gene from *Ginkgo biloba*. Plant Cell Rep..

[47] Sedkova N., Tao L., Rouvière P.E., Cheng Q. (2005). Diversity of carotenoid synthesis gene clusters from environmental enterobacteriaceae strains. Appl. Environ. Microbiol..

[48] Dudareva N., Cseke L., Blanc V.M., Pichersky E. (1996). Evolution of floral scent in *Clarkia*: Novel patterns of S-linalool synthase gene expression in the *C. breweri* flower. Plant Cell.

[49] Nagegowda D.A., Gutensohn M., Wilkerson C.G., Dudareva N. (2008). Two nearly identical terpene synthases catalyze the formation of nerolidol and linalool in snapdragon flowers. Plant J..

[50] Chen F., Tholl D., D’Auria J.C., Farooq A., Pichersky E., Gershenzon J. (2003). Biosynthesis and emission of terpenoid volatiles from *Arabidopsis* flowers. Plant Cell.

[51] Wang J.H., Dudareva N., Bhakta S., Raguso R.A., Pichersky E. (1998). Floral scent production in *Clarkia breweri*. Plant Physiol..

[52] Xiang L., Zhao K.G., Chen L.Q. (2010). Molecular cloning and expression of *Chimonanthus praecox* farnesyl pyrophosphate synthase gene and its possible involvement in the biosynthesis of floral volatile sesquiterpenoids. Plant Physiol. Biochem..

[53] Han Y.J., Wang X.H., Chen W.C., Dong M.F., Yuan W.J., Liu X., Shang F.D. (2014). Differential expression of carotenoid-related genes determines diversified carotenoid coloration in flower petal of *Osmanthus fragrans*. Tree Genet. Genomes.

[54] Yang X.L., Shi T.T., Wen A.L., Wang L.G. (2015). Variance analysis of aromatic components from different varieties of *Osmanthus fragrans*. Dongbei Linye Daxue Xuebao.

[55] Nagegowda D.A., Rhodes D., Dudareva N. (2006). The role of methylerythritol-phosphate pathway in rhythmic emission of volatiles. Biology of Floral Scent.

[56] Mandel M.A., Feldmann K.A., Herrera-Estrella L., Rocha-Sosa M., Leon P. (1996). *CLA1*, a novel gene required for chloroplast development, is highly conserved in evolution. Plant J..

[57] Dudareva N., Andersson S., Orlova I., Gatto N., Reichelt M., Rhodes D., Boland W., Gershenzon J. (2005). The nonmevalonate pathway supports both monoterpene and sesquiterpene formation in snapdragon flowers. Proc. Natl. Acad. Sci. USA.

[58] Hendel-Rahmanim K., Masci T., Vainstein A., Weiss D. (2007). Diurnal regulation of scent emission in rose flowers. Planta.

[59] Pokhilko A., Bou-Torrent J., Pulido P., Rodríguez-Concepción M., Ebenhöh O. (2015). Mathematical modelling of the diurnal regulation of the MEP pathway in *Arabidopsis*. New Phytol..

