# Erratum Xu T. et al. Cloning and Expression Analysis of MEP Pathway Enzyme-encoding Genes in *Osmanthus fragrans.* Genes 2016, *7*, 78

**DOI:** 10.3390/genes8020058

**Published:** 2017-02-02

**Authors:** Chen Xu, Huogen Li, Xiulian Yang, Chunsun Gu, Hongna Mu, Yuanzheng Yue, Lianggui Wang

**Affiliations:** 1 College of Landscape Architecture, Nanjing Forestry University, Nanjing 210037, China; xc127@foxmail.com (C.X.); yangxl339@sina.com (X.Y.); yuanzhengyue@163.com (Y.Y.); 2 Key Laboratory of Forest Genetics & Gene Engineering of the Ministry of Education, Nanjing Forestry University, Nanjing 210037, China; hgli@njfu.edu.cn; 3 Institute of Botany, Jiangsu Province and Chinese Academy of Sciences, Nanjing 210014, China; chunsungu@126.com; 4 College of Horticulture and Gardening, Yangtze University, Jingzhou 434025, China; hongnamu@163.com

The authors wish to make the following correction to their paper [1]. The name of the second author was misspelt. The correct name should be “Huogen Li”. The authors would like to apologize for any inconvenience caused. The change does not affect the scientific results. The manuscript will be updated and the original will remain online on the article webpage.
