# Supplementary material for: Cloning and Expression Analysis of MEP Pathway Enzyme-encoding Genes in Osmanthus fragrans
Source: Genes (Basel). 2016 Sep 29;7(10):78. doi: 10.3390/genes7100078 (PMC5083917; doi:10.3390/genes7100078)
Supplement: Supplementary file 1 [file genes-07-00078-s001.doc]

Supplementary Materials: Cloning and Expression Analysis of MEP Pathway Enzyme-encoding Genes in *Osmanthus fragrans*

Chen Xu, Huogeng Li, Xiulian Yang, Chunsun Gu, Hongna Mu, Yuanzheng Yue and Lianggui Wang

**Table S1.** Primers used for partial cDNA cloning

| **Gene name** | **Primer sequence (5′-3′)** | **PCR condition** |
| --- | --- | --- |
| OfDXS1-F | GATGGCTCTCTTTACACTTGC | 94 °C 3 min, 30 cycles of (94 °C 30 s; 61 °C 30 s; 72 °C 2 min), 72 °C 10 min |
| OfDXS1-R | AGCCAGAGTCCATCCTAAGAC |
| OfDXS2-F | CGGTCCTGATCCATATTGT | 94 °C 3 min, 30 cycles of (94 °C 30 s; 58 °C 30 s; 72 °C 2 min), 72 °C 10 min |
| OfDXS2-R | TAGAGCTTCTTTGGGCCTT |
| OfDXR-F | TGACTGGTAGTAGAGTGACACAC | 94 °C 3 min, 30 cycles of (94 °C 30 s; 61 °C 30 s; 72 °C 2 min), 72 °C 10 min |
| OfDXR-R | CAACTCATACAAGAGCAGGAC |
| OfMCT-F | GCCAAAGCAGTATCTTCCAC | 94 °C 3 min, 30 cycles of (94 °C 30 s; 62 °C 30 s; 72 °C 1 min), 72 °C 7 min |
| OfMCT-R | CAGCAAGTAACAAATCATCAGG |
| OfCMK-F | AGTCAACTGTGTTCATCTTC | 94 °C 3 min, 30 cycles of (94 °C 30 s; 51 °C 30 s; 72 °C 2 min), 72 °C 10 min |
| OfCMK-R | GACAACAAAGTACAACAGTC |
| OfMDS-F | ATGGCGACTTCAAACCAC | 94 °C 3 min, 30 cycles of (94 °C 30 s; 58 °C 30 s; 72 °C 1 min), 72 °C 7 min |
| OfMDS-R | CCTCGTAAGGAGAACCACT |
| OfHDS-F | AACGGTCGGGATTTGGAGTTG | 94 °C 3 min, 30 cycles of (94 °C 30 s; 65 °C 30 s; 72 °C 3 min), 72 °C 10 min |
| OfHDS-R | GCCCTTCTGCCCATAAACTTA |
| OfHDR1-F | TGTGGACTACTGTTGAAAAGC | 94 °C 3 min, 30 cycles of (94 °C 30 s; 60 °C 30 s; 72 °C 1 min), 72 °C 7 min |
| OfHDR1-R | ATTTTCCACTCCATACTTGCG |
| OfHDR2-F | TTGGAGCTGCTGTGGATGA | 94 °C 3 min, 30 cycles of (94 °C 30 s; 57 °C 30 s; 72 °C 1 min), 72 °C 7 min |
| OfHDR2-R | CGAGCCTCTGCTATTTCTTGTA |
| OfIDI-F | CGCCACTGATTCCGGTATG | 94 °C 3 min, 30 cycles of (94 °C 30 s; 62 °C 30 s; 72 °C 1 min), 72 °C 7 min |
| OfIDI-R | TCGGCTGCTTTGCTCAGAG |

**Table S2. Primers used for RACE reaction**

| **Usage** | **Gene name** | **Primer sequence (5′-3′)** | **PCR condition** |
| --- | --- | --- | --- |
| 3′ RACE  Outer PCR | OfDXS1-3′OF | TTTGTGCAATCTACTCGTCCTTC | 94 °C 3 min,  20 cycles of (94 °C  30 s; 55 °C 30 s;  72 °C 2 min),  72 °C 10 min |
|  | OfDXS2-3′OF | TCTGTGCCATCTATTCGTCATTC |  |
|  | OfDXR-3′OF | AGGTCCATTTGTACTTCCTCTTG |  |
|  | OfMCT-3′OF | AATTTGCATTGCCCGGAAAGGAG |  |
|  | OfCMK-3′OF | TCCATTGGTTCTCATAAAGCCTC |  |
|  | OfMDS-3′OF | TTACACTTCCAGCAGAAACCTTC |  |
|  | OfHDS-3′OF | CGATGAATCCCAAGAAGAGTTTG |  |
|  | OfHDR1-3′OF | GGACTACTGTTGAAAAGCACAAG |  |
|  | OfHDR2-3′OF | ATGAAGAGACTGTAGCAACTGCT |  |
|  | OfIDI-3′OF | CTCTTGGATGAACTTGGTATTCC |  |
| 3′ RACE  Inner PCR | OfDXS1-3′IF | AGTGATGGCTCCTTCTGATGAGGCTGAG | 94 °C 3 min,  30 cycles of (94 °C  30 s;60 °C 30 s;  72 °C 1.5 min),  72 °C 10 min |
|  | OfDXS2-3′IF | TGTAGCATACATGGCTTGTTTGCCCAAC |  |
|  | OfDXR-3′IF | GTGGTGCTTTTAGGGATTTGCCTGCTGA |  |
|  | OfMCT-3′IF | GTTCCTGCCAAAGCTACTATCAAAGAGG |  |
|  | OfCMK-3′IF | ATTGCCGCAGGTCGAGGACAGTATGATG |  |
|  | OfMDS-3′IF | ATCGAGGCTGTGAAGCTCACTCAGATGG |  |
|  | OfHDS-3′IF | CACAGGGACGATTTAGTCATTGGTGCTG |  |
|  | OfHDR1-3′IF | GTGTGCGATTACATTTTGGGTGGTGAAC |  |
|  | OfHDR2-3′IF | TGTTGAAGGGAGAAACAGAGGAGATTGG |  |
|  | OfIDI-3′IF | GATTACCTGCTCTTCATTGTTCGGGATG |  |
| 5′ RACE  Outer PCR | OfDXS1-5′OF | GATATGACTGGTGCCCAACATCC | 94 °C 3 min,  20 cycles of (94 °C  30 s; 55 °C 30 s;  72 °C 2 min),  72 °C 10 min |
|  | OfDXS2-5′OF | GACGAATAGATGGCACAGAACGG |  |
|  | OfDXR-5′OF | TCCCGTAACTACTGTGACAGCGT |  |
|  | OfMCT-5′OF | ACTTCAAGACCTTCCCTGTTCAC |  |
|  | OfCMK-5′OF | ACATGAAAGAGCGACGCCAAATC |  |
|  | OfMDS-5′OF | GCCTCCTTGTGTGGGCTTACTTT |  |
|  | OfHDS-5′OF | CGCTTCTTTCTTTCCCTGCACTG |  |
|  | OfHDR1-5′OF | ACGTTGTGCCGGTATTGAGACCT |  |
|  | OfHDR2-5′OF | TCTCCTCTGTTTCTCCCTTCAAC |  |
|  | OfIDI-5′OF | GCCGACGGCATTTCTAAGTCAAC |  |
| 5′ RACE  Inner PCR | OfDXS1-5′IF | TTGATCGTGTCCAAAAGAGGAGTCGGAG | 94 °C 3 min,  30 cycles of (94 °C  30 s; 60 °C 30 s;  72 °C 1.5 min),  72 °C 10 min |
|  | OfDXS2-5′IF | GTTCCACCACCCATTGCGGCATGAATAG |  |
|  | OfDXR-5′IF | TCACCTGGTCGGCAAGAAGAGTCACATT |  |
|  | OfMCT-5′IF | CCCAATAAGCCAACCATCCTTTAGGACC |  |
|  | OfCMK-5′IF | TCATACGCTACCTCCACTTTTCTGCCAG |  |
|  | OfMDS-5′IF | TATATCTGGAAGCCCCAATGCCCCCAAT |  |
|  | OfHDS-5′IF | CTCACTACCAAGAGCCACATTTCCAACC |  |
|  | OfHDR1-5′IF | TTACAGGACCTTCTGGTAGCCAGTTCTC |  |
|  | OfHDR2-5′IF | GTTCACCACCCAAAATGTAATCGCACAC |  |
|  | OfIDI-5′IF | GCTTAATACCACCCTCACCAGCATCTGC |  |

**Table S3. Primers used for qRT-PCR**

| **Gene name** | **Primer sequence (5**′**-3**′**)** | **Amplification length** |
| --- | --- | --- |
| OfDXS1-qF | AGTCACCGAGAAAGGCAGA | 239 bp |
| OfDXS1-qR | GGAAGCGACGCAAGAAAA |  |
| OfDXS2-qF | GGGCATTTGATGTAGCATA | 208 bp |
| OfDXS2-qR | ATCCTTCCCTTACCAATCT |  |
| OfDXR-qF | CCTCAAATTCCAAGGTGGGT | 219 bp |
| OfDXR-qR | GGATTCTCCGCAACTATGTC |  |
| OfMCT-qF | CCTAAAGGATGGTTGGCTTAT | 214 bp |
| OfMCT-qR | TGATACATCGTCGGTTACTTC |  |
| OfCMK-qF | GTAGTAGTAATGCCGCCACA | 198 bp |
| OfCMK-qR | ATGGAACGTCAAAGGGTAGA |  |
| OfMDS-qF | GCTTGCTTTACACGGAACT | 200 bp |
| OfMDS-qR | CCGATAATCAGCGGATAAC |  |
| OfHDS-qF | ACGAAGCCATTACCCTACA | 214 bp |
| OfHDS-qR | TTGCTGCATGAACTCTGCT |  |
| OfHDR1-qF | GCTAGAAAACAATTCCCAA | 159 bp |
| OfHDR1-qR | TACCACAACATCACCCTTA |  |
| OfHDR2-qF | ACAATCGGAAGGGGTTT | 195bp |
| OfHDR2-qR | TTCTCGCCTCGTAAGCA |  |
| RAN-qF | AGAACCGACAGGTGAAGGCAA | 117 bp |
| RAN-qR | TGGCAAGGTACAGAAAGGGCT |  |
| RPB2-qF | CACCAAGCAAAGGACCAGCAAG | 216 bp |
| RPB2-qR | TCACCAGGGAGAAGAGGATCAAGTA |  |
| ACT-qF | CCCAAGGCAAACAGAGAAAAAAT | 143 bp |
| ACT-qR | ACCCCATCACCAGAATCAAGAA |  |

**Table S4. Conserved domain analysis for MEP genes**

| **Gene** | **Size (ORF)** | **Characteristic domain** |
| --- | --- | --- |
| OfDXS1 | 2172 bp | **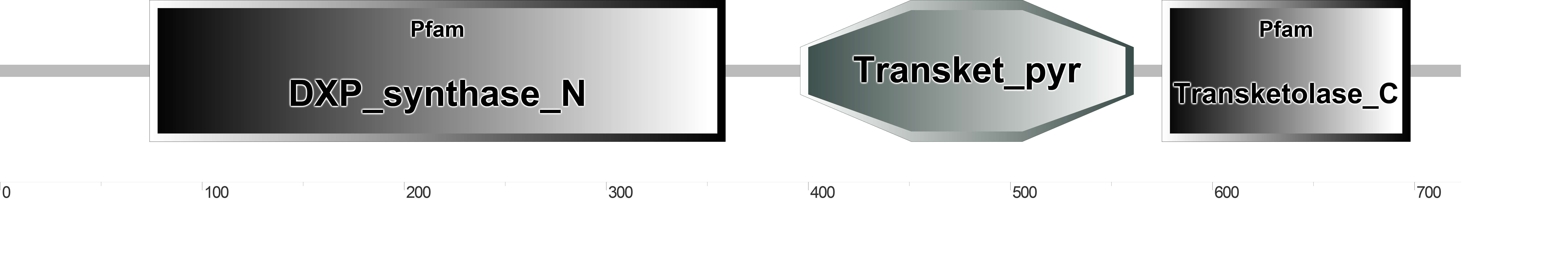** |
| OfDXS2 | 2148 bp | **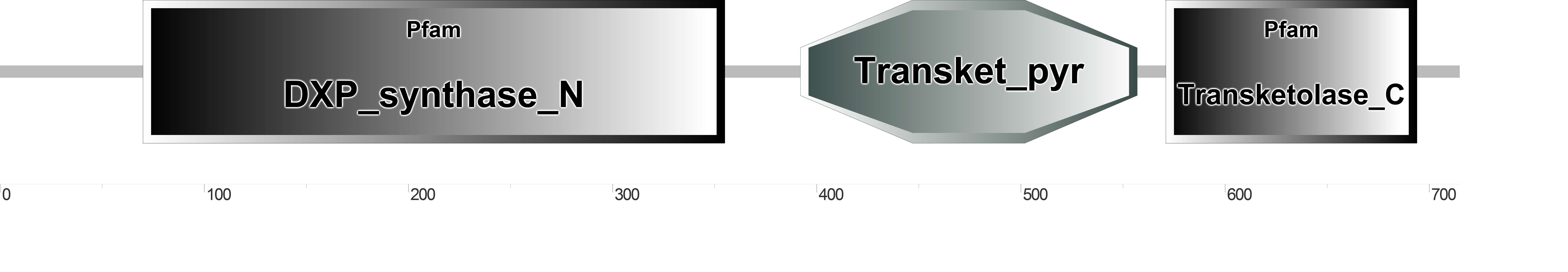** |
| OfDXR | 1425 bp | **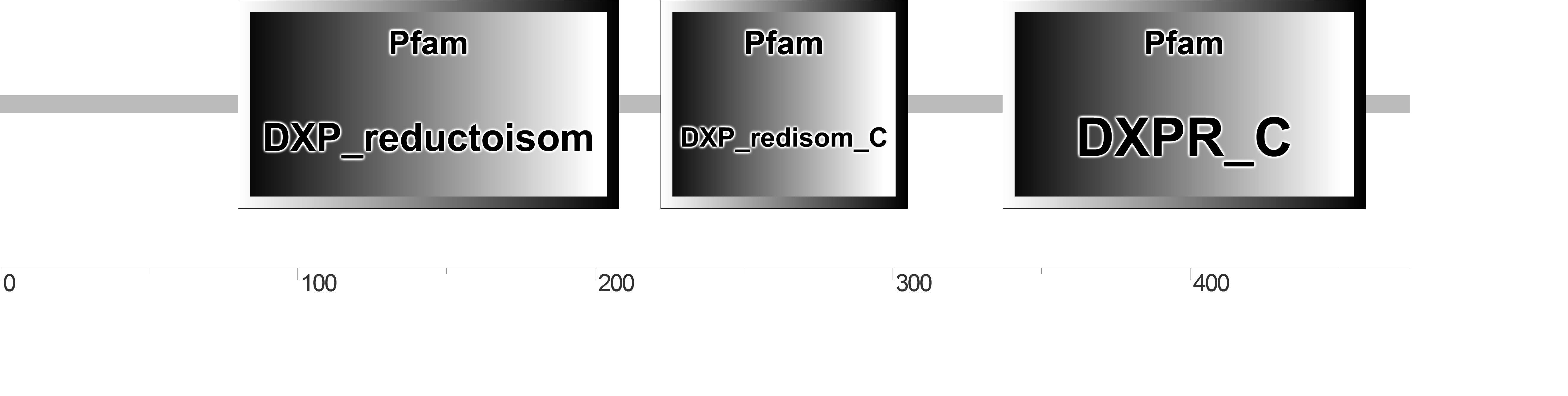** |
| OfMCT | 939 bp | **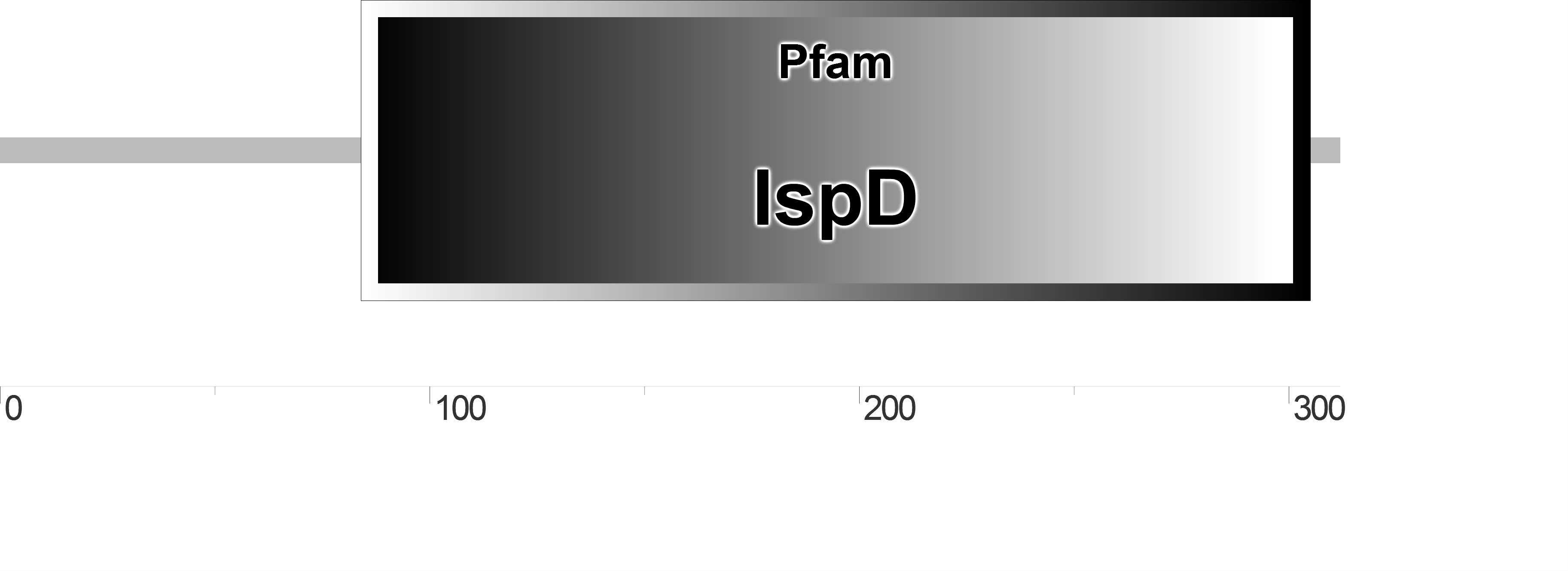** |
| OfCMK | 1206 bp | **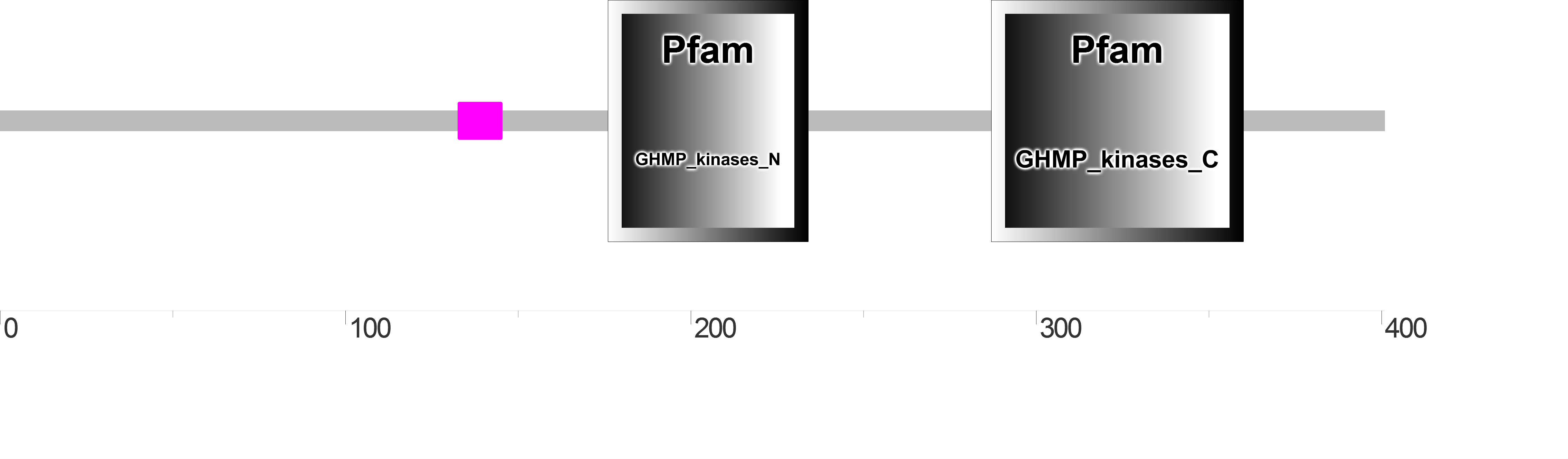** |
| OfMDS | 702 bp | **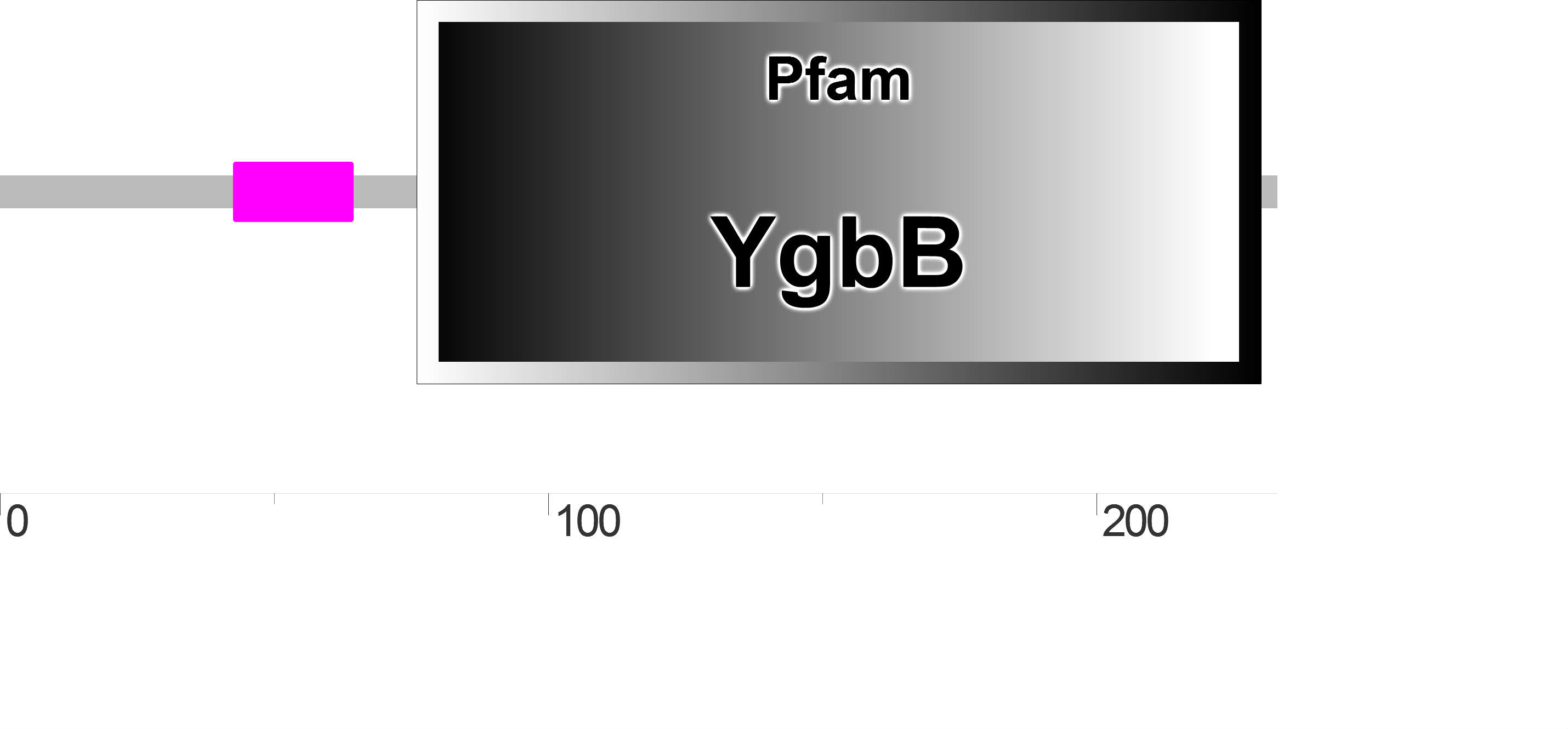** |
| OfHDS | 2229 bp | **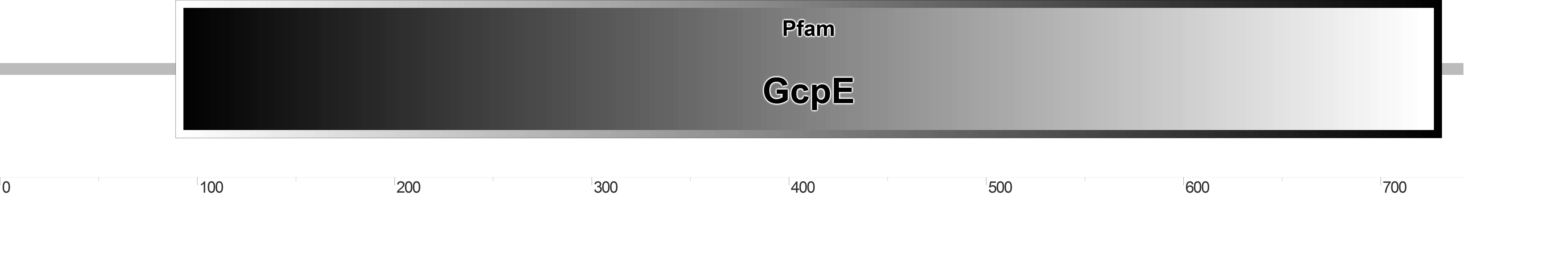** |
| OfHDR1 | 1386 bp | **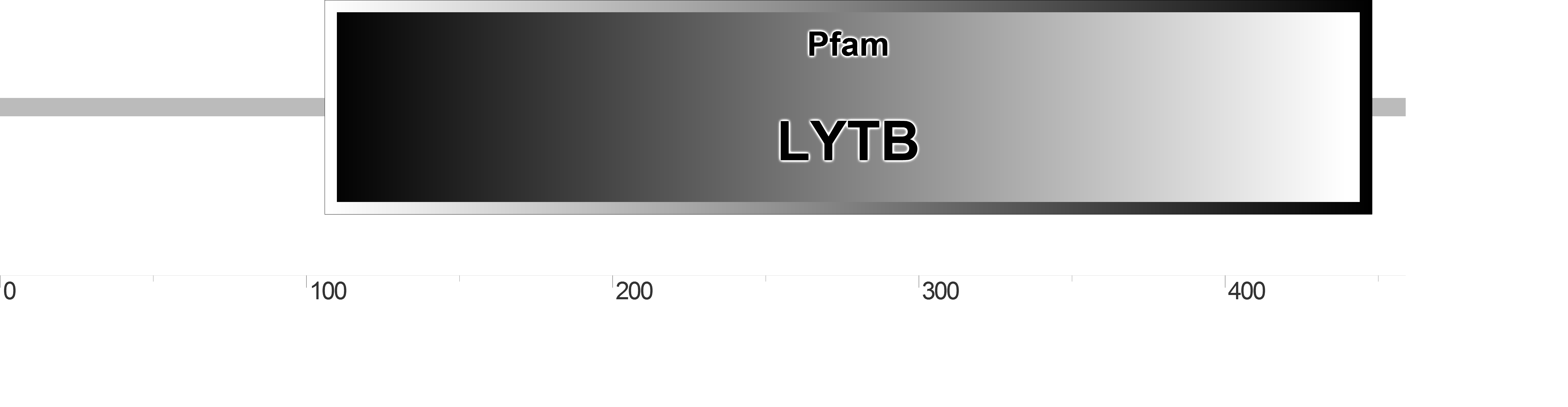** |
| OfHDR2 | 1380 bp | 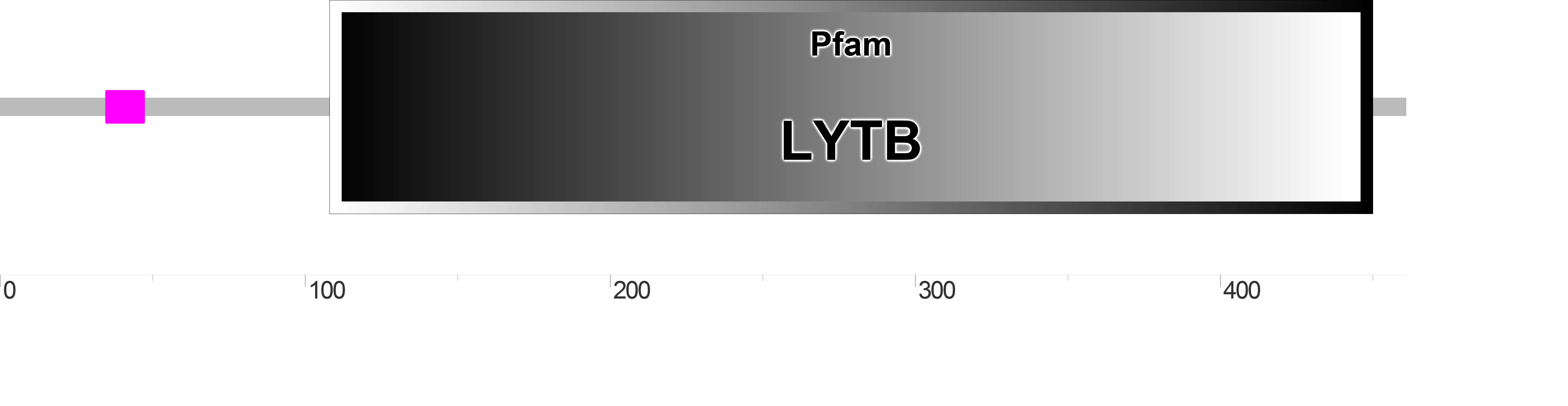 |
| OfIDI | 708 bp | **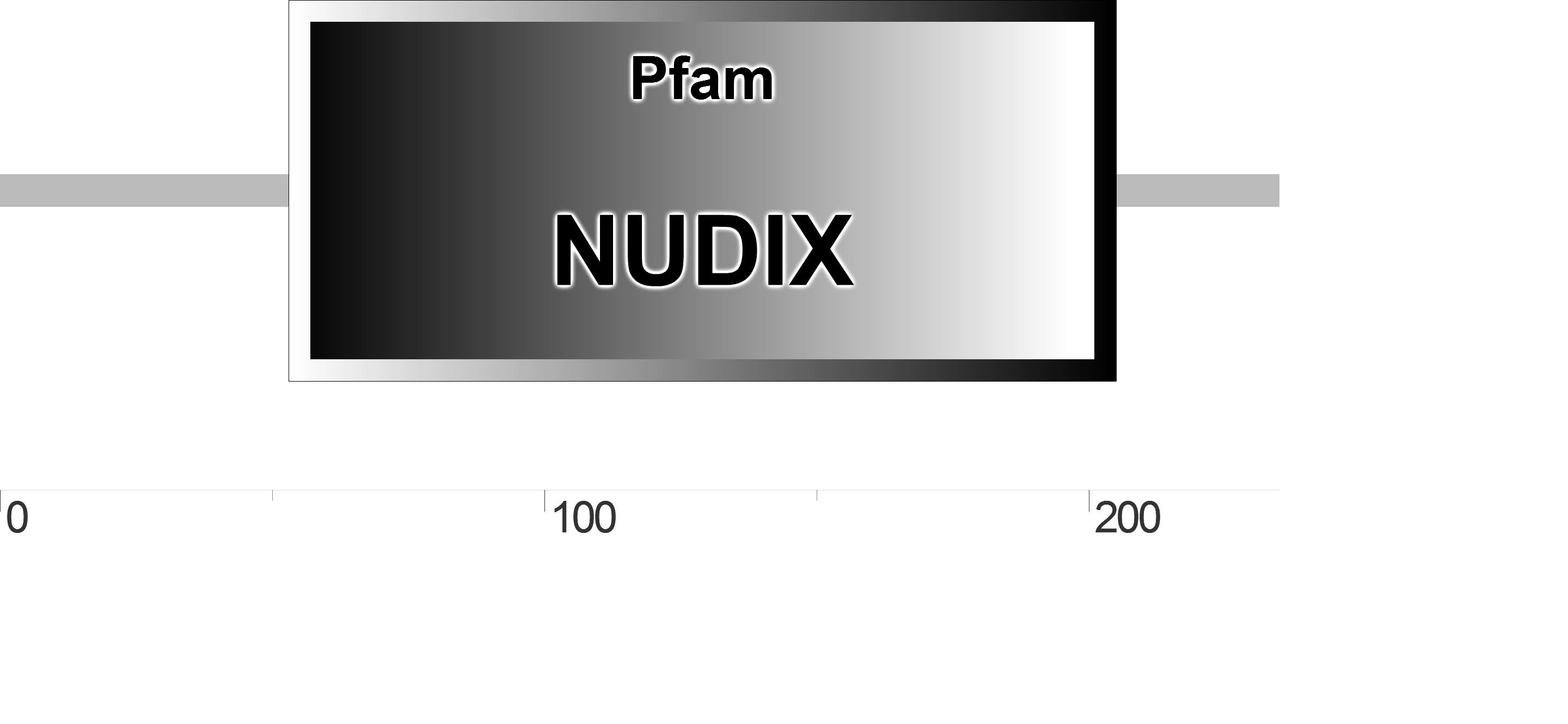** |
